# Influence of Magnetite Nanoparticles Shape and Spontaneous Surface Oxidation on the Electron Transport Mechanism

**DOI:** 10.3390/ma14185241

**Published:** 2021-09-12

**Authors:** Adrian Radoń, Mariola Kądziołka-Gaweł, Dariusz Łukowiec, Piotr Gębara, Katarzyna Cesarz-Andraczke, Aleksandra Kolano-Burian, Patryk Włodarczyk, Marcin Polak, Rafał Babilas

**Affiliations:** 1 Łukasiewicz Research Network—Institute of Non-Ferrous Metals, Sowinskiego 5 St., 44-100 Gliwice, Poland; aleksandra.kolano-burian@imn.gliwice.pl (A.K.-B.); patryk.wlodarczyk@imn.gliwice.pl (P.W.); marcin.polak@imn.gliwice.pl (M.P.); 2 Faculty of Mechanical Engineering, Silesian University of Technology, Konarskiego 18 a St., 44-100 Gliwice, Poland; dariusz.lukowiec@polsl.pl (D.Ł.); katarzyna.cesarz-andraczke@polsl.pl (K.C.-A.); rafal.babilas@polsl.pl (R.B.); 3 A. Chelkowski Institute of Physics, University of Silesia, 75 Pułku Piechoty 1A St., 41-500 Chorzów, Poland; mariola.kadziolka-gawel@us.edu.pl; 4 Institute of Physics, Czestochowa University of Technology, al. Armii Krajowej 19, 42-200 Czestochowa, Poland; gebara.piotr@wip.pcz.pl

**Keywords:** magnetite, maghemite, electrical conductivity, surface oxidation

## Abstract

The spontaneous oxidation of a magnetite surface and shape design are major aspects of synthesizing various nanostructures with unique magnetic and electrical properties, catalytic activity, and biocompatibility. In this article, the roles of different organic modifiers on the shape and formation of an oxidized layer composed of maghemite were discussed and described in the context of magnetic and electrical properties. It was confirmed that Fe_3_O_4_ nanoparticles synthesized in the presence of triphenylphosphine could be characterized by cuboidal shape, a relatively low average particle size (9.6 ± 2.0 nm), and high saturation magnetization equal to 55.2 emu/g. Furthermore, it has been confirmed that low-frequency conductivity and dielectric properties are related to surface disordering and oxidation. The electric energy storage possibility increased for nanoparticles with a disordered and oxidized surface, whereas the dielectric losses in these particles were strongly related to their size. The cuboidal magnetite nanoparticles synthesized in the presence of triphenylphosphine had an ultrahigh electrical conductivity (1.02 × 10^−4^ S/cm at 10 Hz) in comparison to the spherical ones. At higher temperatures, the maghemite content altered the behavior of electrons. The electrical conductivity can be described by correlated barrier hopping or overlapping large polaron tunneling. Interestingly, the activation energies of electrons transport by the surface were similar for all the analyzed nanoparticles in low- and high-temperature ranges.

## 1. Introduction

The modification of the surface of nanoparticles (NPs) plays an essential role in the synthesis of materials with controlled properties. The formation of functionalized nanoparticles with core–shell nanostructures are widely studied, especially in medical and catalytic applications [1,2,3,4]. This procedure allows one to change the properties of nanoparticles, especially to increase their reactivity, stability, and even biocompatibility. For many different nanostructures, the formation of particles with a core–shell structure results in highly efficient gas sensors synthesis. For example, Au@NiO nanoparticles are much more sensitive to ethanol than pristine NiO, which is related to the effect of the electronic and chemical sensitization of Au [5]. The synergetic effects of two different oxides on acetone sensitivity are also observed in systems such as Fe_3_O_4_@Co_3_O_4_ and are associated with the formation of the heterojunction [6]. In addition, the core–shell combination of nanoparticles and the functionalization process can be used to prepare materials with high catalytic activity. Sappino et al. [7] demonstrated that Fe_3_O_4_@SiO_2_ nanoparticles with a core–shell structure could be successfully functionalized to obtain stable and removable amino alcohol catalysts that can be used for the asymmetric addition of diethylzinc to aromatic aldehydes. The functionalization of Fe_3_O_4_@SiO_2_ nanoparticles has also been successfully used to prepare nanocatalysts functionalized by alkylsulfonic and butylcaboxylic acids, which were used by Ingle et al. for the production of biofuels from sugarcane straw [8].

On the other hand, magnetite nanoparticles functionalized with polymers, such as polylactic acid, cyclodextrins, amine-terminated poly(propylene glycol), and polyethylene glycol, can be used as magnetic resonance imaging agents and efficient drug delivery systems [9,10]. Therefore, understanding the functionalization mechanisms and the influence of surface modification on the properties of magnetite nanoparticles is essential in various fields of research. Recently, the role of the dissolution of the surface of oxidized magnetite nanoparticles in catalytic activity and magnetoelectric properties was discussed [11]. It has been shown that chemical treatment of magnetite nanoparticles synthesized by the co-precipitation method with malonic acid results in surface reorganization, dissolution of the oxidized layer, and spontaneous functionalization with an organic acid. Moreover, this process reduces the catalytic activity and increases the electrical conductivity of these nanoparticles 10 times.

The oxidation of magnetite to maghemite can occur spontaneously, or it can take place under certain conditions. This process can occur even during the synthesis; therefore, the synthesis should be carried out in anaerobic conditions [12]. The oxidation of a magnetite surface results in phase transformation, in which magnetite is transformed into maghemite [13]. In the magnetite structure, the tetrahedral sites (A-sites) are occupied by Fe^3+^ ions and the octahedral sites (B-sites) are occupied by Fe^3+^ and Fe^2+^ ions, whereas in maghemite, all Fe^2+^ ions convert into Fe^3+^ ions with the formation of iron vacancies (2.5 per 21.5 Fe^3+^ ions in the unit cell) as a result of Fe_3_O_4_ NPs oxidation [4,14]. The magnetic response under AC-fields of magnetite and maghemite nanoparticles of the same size is also different, which has been confirmed by Morales et al. [15]. Therefore, even though both iron oxides exhibit ferrimagnetic properties at room temperature, their electronic properties must be different, which should be related to the changes in electron transport. The electrical properties of pure magnetite nanoparticles have been discussed and described in detail in the previous work [16]. In the pure Fe_3_O_4_ phase, two different models were used to describe the behaviors of electrons at different temperatures and frequency ranges.

Moreover, it was confirmed that the energy of tunneling of small polarons decreases with increasing temperature, and for the high-temperature regions, it may occur spontaneously. Interestingly, the electron behavior in magnetite is strongly related to the structure, and even Drude-like behavior can be observed for this type of material [17]. The Drude model uses the kinetic theory of gases to explain electrons’ movement in, for example, metals and heavy fermionic materials [18]. Above that, the changes in dielectric properties have been observed for various mixed ferrites such as barium ferrite–zinc ferrite and Sr_2_Cu_2_Fe_12_O_22_–zinc ferrite systems, which indicates that the presence of other structures can drastically change the behavior of electrons in this type of material [19,20].

Despite that, there is a lack of knowledge of how the surface modification can change magnetic and dielectric properties of magnetite nanoparticles, especially charge carriers motions and electric energy storage properties. Those, in turn, may contribute to the development of highly sensitive and stable toxic gas sensors [21]. Therefore, this study investigated the influence of magnetite crystallization on spherical and cuboidal nanoparticles and the disordered and oxidized magnetite surface on the behavior of electrons in magnetite nanoparticles. Oxidized magnetite nanoparticles with various γ-Fe_2_O_3_ contents and cuboidal shapes were synthesized by the controlled co-precipitation method, and the maghemite presence was determined by Mössbauer spectroscopy. The mechanism of electrical conductivity in magnetite with different shapes as well as surface disordering and oxidation was proposed and discussed. 

## 2. Materials and Methods

### 2.1. Synthesis of Magnetite Nanoparticles in a Hydrophobic–Hydrophilic Environment

The synthesis of magnetite nanoparticles was performed in a hydrophobic–hydrophilic environment, in which the upper layer was hexadecane, protecting the formed nanoparticles against contact with atmospheric oxygen. First, 20 mmol of FeCl_3_ (anhydrous) and 10 mmol of FeSO_4_·7H_2_O were dissolved in a stock solution with 50 mL of water and 50 mL of hexadecane, respectively. Then, 5 mmol of an organic modifier, i.e., ethylenediamine (en), chloramine T (ClT), or triphenylphosphine (PPh_3_), was added to the as-prepared solution with constant stirring. It was observed that the PPh_3_ was dissolved only in the hexadecane layer due to its low solubility in water. Next, the solution was heated up to 328 K in an ultrasonic water bath during a continuous sonication process. Finally, a solution with 80 mL of water and 200 mmol of NaOH was added dropwise to the reaction mixture. The black precipitate was sonicated in a post-reaction solution throughout the process. The powder was collected by filtration, washed twice with water, ethanol, and acetone and then dried at 333 K for 2 h. The obtained samples were marked as Fe_3_O_4_–en NPs, Fe_3_O_4_–ClT NPs, and Fe_3_O_4_–PPh_3_ NPs, depending on the modifier used for the synthesis. The same synthesis method was used to prepare Fe_3_O_4_ NPs without using any additional organic modifier. 

### 2.2. Materials Characterization

The phase purity and crystal structure of the synthesized nanoparticles were described by X-ray diffraction (XRD). The diffractograms were collected using a Rigaku MiniFlex 600 with a copper tube Cu Kα (*λ* = 0.15406 nm) and a D/teX Ultra silicon strip detector (Rigaku Corporation, Tokyo, Japan). The tube voltage was 40 kV, and the current was 15 mA. The measurements were performed with a step width of 0.02° in the scan range from 10° to 90°. The phase analysis was performed using a dedicated Rigaku PDXL software suite. Fourier-transform infrared (FTIR) spectroscopy was used to determine the presence of organic modifiers on the surface of magnetite nanoparticles and confirm the presence of maghemite on their surface. FTIR spectra were recorded in the infrared transmission mode with a range of 4000–400 cm^−1^ using a Nicolet 6700/8700 FTIR spectrometer (Thermo Fisher Scientific, Waltham, MA, USA) and the KBr pellet method. The transmission electron microscope (TEM) micrographs of the magnetite nanoparticles deposited on the surface of a copper grid with a carbon film were collected using S/TEM TITAN 80-300 (FEI Company, Eindhoven, The Netherlands). The average nanoparticles size (*D_av_*) was calculated based on the analysis of TEM micrographs. For this purpose, the size of nanoparticles was measured for at least 100 different particles and at least 5 different micrographs. The *D_av_* value was calculated as a mean value. The magnetic properties of magnetite nanoparticles were determined by a Quantum Design VersaLab (Quantum Design, San Diego, CA, USA) cryogen-free vibrating-sample magnetometer (VSM). To determine the presence of an oxidized surface and to confirm the existence of the magnetite structure, the ^57^Fe Mössbauer spectra (MS) were recorded using the MS96 spectrometer (Tampa, FL, USA) with a ^7^Co: Rh source (activity ~ 25mCi), the linear arrangement of the ^57^Co source, absorber and detector and a multichannel analyzer with 1024 channels. A metallic α-Fe foil absorber was used to calibrate velocity and isomer shift. Spectral analysis was performed using WMOSS software (Ion Prisecaru, WMOSS4 Mössbauer Spectral Analysis Software, 2009–2016). Measurements of dielectric spectra (complex permittivity and dielectric losses) and electrical conductivity were performed for compressed samples in the form of discs with a uniform diameter of 10 mm at a compression pressure of 30 bar. Measurements were performed using a Concept 81 dielectric spectrometer equipped with a Novo-cool temperature control system and an Alpha analyzer (Novocontrol, Montabaur, Germany) in the frequency range from 0.01 to 10 MHz and in the temperature range of 173–363 K with ΔT equal to 10 K. 

## 3. Results and Discussion

### 3.1. Structural Analysis and Magnetic Properties of Magnetite Nanoparticles 

**Figure 1:**
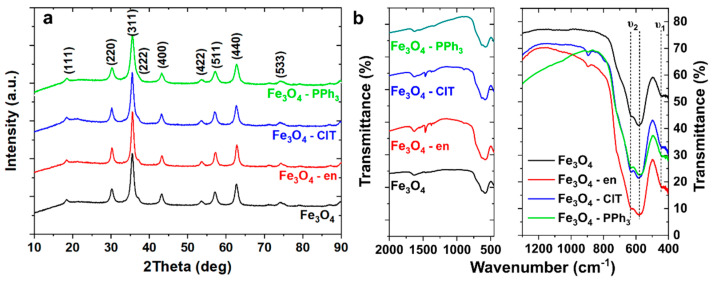
Structural analysis of magnetite nanoparticles: (**a**) X-ray diffraction patterns of magnetite nanoparticles synthesized without (Fe_3_O_4_) and with the presence of ethylenediamine (Fe_3_O_4_–en), chloramine T (Fe_3_O_4_–ClT), and triphenylphosphine (Fe_3_O_4_–PPh_3_); (**b**) Fourier-transform infrared spectra of the same samples. The left panel presents the spectra in a wide frequency range, and the right panel shows the spectra in a narrow frequency range, in which characteristic vibrations from Fe–O bonds (*υ*_1_ and *υ*_2_) occurred.

**Figure 2:**
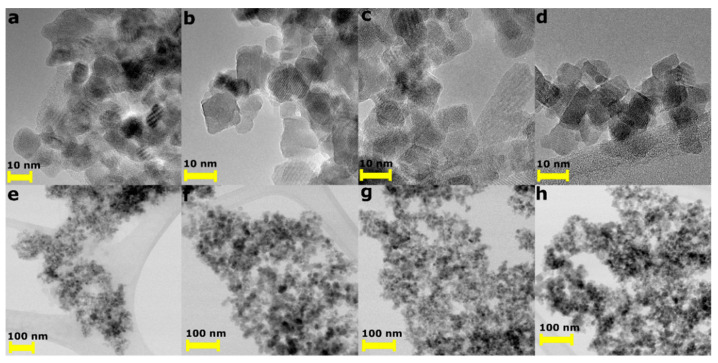
Transmission electron microscopy images of magnetite nanoparticles synthesized without (**a**,**e**) and with the presence of en (**b**,**f**), ClT (**c**,**g**), and PPh_3_ (**d**,**h**).

The XRD patterns recorded for synthesized magnetite nanoparticles using various organic modifiers are shown in Figure 1a. As one can see, all patterns can be very well described using Miller indices characteristic to the magnetite phase (Fd-3m; *a* = *b* = *c* = 0.8375 nm; *α* = *β* = *γ* = 90°). Based on the measured XRD patterns, average crystallite size and intrinsic strain were calculated using the Halder–Wagner method, which was previously used to determine the size of crystallites of magnetite and other ferrites [22,23,24]. Intrinsic strain in nanomaterials is prevalent and can be determined by analyzing the broadening of diffraction peaks. This broadening in nanomaterials is related to the fine size and the existence of strain, which is related to the high concentration of defects such as dislocations, point defects, stacking faults, and grain boundaries [25]. Therefore, strain analysis can provide helpful information on the defects concentration in magnetite nanoparticles. The results of the analysis are presented in Table 1. As can be seen, the use of chloramine T and ethylenediamine resulted in the synthesis of nanoparticles with an average crystallite size (*D_H-W_*) equal to or greater than 10 nm, while the lowest *D_H-W_* value equal to 8.04 nm was found for nanoparticles synthesized with the use of PPh_3_. Interestingly, the analysis of intrinsic strain showed that the highest *ε* value was obtained for nanoparticles synthesized with PPh_3_ and the lowest value (undetectable) was achieved for nanoparticles synthesized without organic modifiers. The presence of the strain in nanoparticles can be related to the numerous factors such as ultrafine size, different shapes, highly disordered surface, and presence of vacancies [26,27]. To understand these changes in *D_H-W_* and *ε*, the analyses of the FTIR spectra (presented in Figure 1b) and the TEM images (presented in Figure 2) were performed. 

The FTIR spectra over a wide range of wavenumbers (left panel in Figure 1b) confirmed that Fe_3_O_4_ NPs and Fe_3_O_4_–PPh_3_ NPs were pure in the context of the presence of hexadecane and PPh_3_. Accordingly, PPh_3_ molecules only changed the average crystallite size and intrinsic strain in the synthesis step by interacting with the crystallized nanoparticles without spontaneously functionalizing them. In the case of Fe_3_O_4_–en NPs and Fe_3_O_4_–ClT NPs, the presence of C–H bonds was confirmed in the FTIR spectra. Therefore, both organic modifiers changed the structure and morphology of nanoparticles and functionalize their surface. Probably negatively charged molecules interacted with iron ions and could not be removed even when washing samples [28]. The narrow range of wavenumbers (right panel in Figure 1b) showed the two characteristic vibrations of Fe–O bonds (marked as *υ*_1_ and *υ*_2_). These vibrations are also listed in Table 1. For bulk magnetite, the *υ*_1_ vibrations occurred at much lower wavenumbers (about 375 cm^−1^); however, for nanoparticles, peak shifts to higher wavenumbers can occur, which is related to the fine size of the particles [29]. The dual nature of the *υ*_2_ is related to the oxidized surface in which maghemite is formed. For this phase, vibrational mode with the T_2_ symmetry occurs, whereas only one vibration mode with T_1u_ symmetry at ±600 cm^−1^ can be observed for pure magnetite nanoparticles [30].

The analysis of TEM images confirmed that all nanoparticles had an ultrafine size; however, their size distribution was broad (*D_av_* in Table 1). This is characteristic of magnetite nanoparticles synthesized by the co-precipitation method. In this method, it is challenging to control the growth of Fe_3_O_4_ and synthesize uniform monodisperse nanoparticles. Moreover, as shown in Figure 2, all nanoparticles were strongly agglomerated, and only the Fe_3_O_4_ NPs had a near-spherical shape. Other samples crystallized into cuboidal forms. Moreover, their surface was strongly disordered, which is consistent with the measurements of intrinsic strain. For the Fe_3_O_4_–en and Fe_3_O_4_–PPh_3_ nanoparticles, ε was the highest, and after analysis of the TEM micrographs, it can be connected primarily with the highly disordered surface.

**Figure 3:**
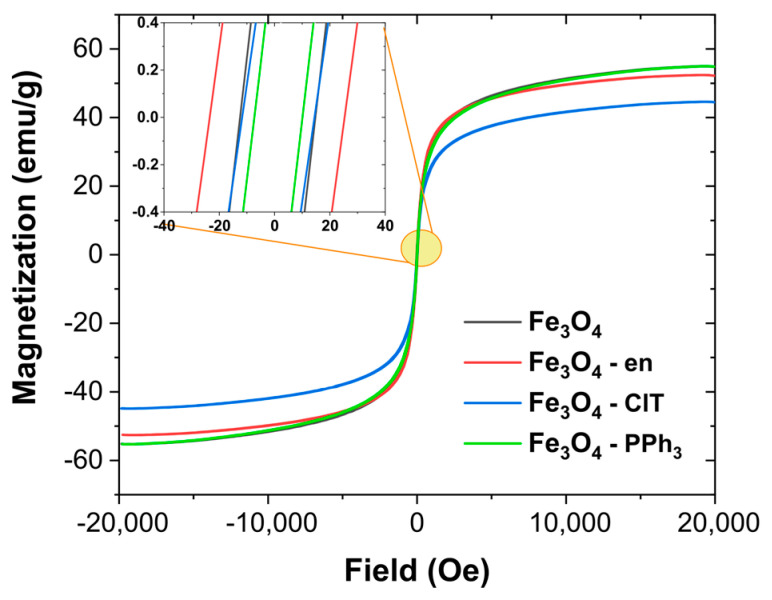
Magnetization curves obtained by a vibrating-sample magnetometer. The inset shows the enlarged area near the coercive field.

As presented above, the structural analysis clearly showed that the average crystallite size, shape, and surface functionalization were different for all synthesized samples. Therefore, their magnetic properties should be different. Interestingly, the *D_H-W_* values were similar for all samples (between 8.04 and 11.8 nm). Therefore, the changes in magnetic behavior could be related mainly to differences in the surface of nanoparticles. The recorded VSM curves are shown in Figure 3, and the parameters obtained from the curves, i.e., saturation magnetization (*M_s_*), remanence (*M_r_*), coercivity (*H_c_*), and *M_r_*/*M_s_* ratio, are summarized in Table 2. It can be noticed that Fe_3_O_4_ NPs and Fe_3_O_4_–PPh_3_ NPs had similar magnetic properties, especially the *M_s_* value. For nanoparticles synthesized using PPh_3_, the *M_r_* and *H_c_* values were lower than for nanoparticles synthesized without organic modifiers, which confirmed that PPh_3_ plays a crucial role in synthesizing Fe_3_O_4_ NPs with good superparamagnetic properties. Lower *M_s_* values were recorded for nanoparticles synthesized in the presence of ClT and en. For Fe_3_O_4_–ClT NPs, *H_c_* was much slower than for Fe_3_O_4_–en NPs, but the *M_s_* value was only 44.75 emu/g, which is probably related to the presence of a highly oxidized surface.

**Figure 4:**
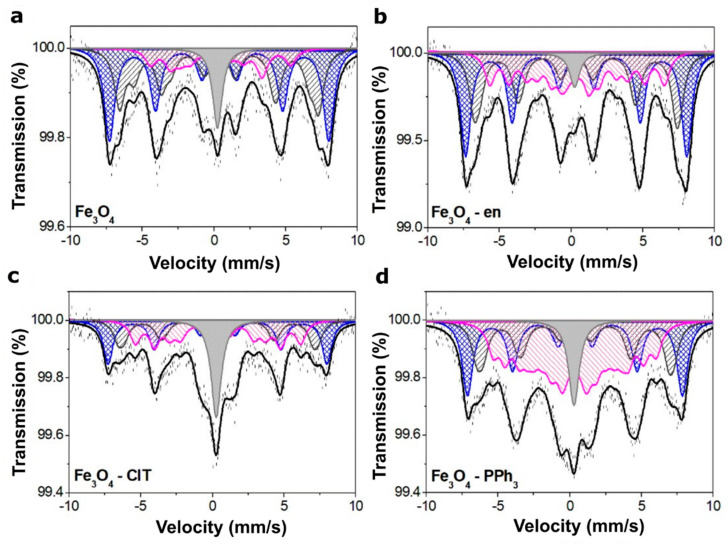
Mössbauer spectra of magnetite nanoparticles synthesized without (**a**) and with the presence of different organic modifiers: en (**b**), ClT (**c**), and PPh_3_ (**d**). The contribution of the γ-Fe_2_O_3_ is displayed in blue, and that of the Fe_3_O_4_ is displayed in dark grey; the sextets connected with the magnetic interaction between nanoparticles are displayed in magenta, and the contribution of superparamagnetic fine particles is displayed in light gray.

The analysis of Mössbauer spectra (MS) can provide very useful information on the oxidation of a magnetite surface. The same analysis cannot be performed with the XRD and TEM methods because of minor differences between the maghemite and magnetite unit cell parameters. To confirm the presence of maghemite on the surface of magnetite nanoparticles, the MS at room temperature were recorded for all samples and are presented in Figure 4. These spectra revealed asymmetrical six-line patterns with broad lines and were fitted using five to seven magnetic sextets and one single line. In turn, the obtained hyperfine parameters are summarized in Table 3. The full line widths at half maximum for all components were equal to 0.85 mm/s. The observed broad line width of the sextets confirmed the superparamagnetic size of the magnetite nanoparticles.

Note maghemite is cation-deficient spinel with Fe^3+^ on both the octahedral and tetrahedral sites. The contribution from Fe^3+^ on both sites of magnetically ordered maghemite led to the MS consisting of two sextets with similar hyperfine magnetic fields, essentially zero quadrupole splitting and isomer shifts characteristic for Fe^3+^ in tetrahedral and octahedral coordination. However, room-temperature MS of maghemite appeared as a single sextet (S1), which is evident since the contributions from Fe^3+^ ions at both sites of the maghemite spinel structure had close values. Sextets S2 and S3 represented iron ions located in the tetrahedral and octahedral sites in the Fe_3_O_4_ structure, respectively. Broader lines and smaller hyperfine fields can be a result of some non-stoichiometric of the crystals.

The MS of bulk magnetite is different than for nanoparticles, which is associated with a high contribution from the surface of nanoparticles, which is very important at the nanoscale and cannot be neglected. Furthermore, in the case of nanoparticles, much more sextets can be observed. Some are related to the maghemite formation on the magnetite surface, and others are associated to the external region and the transition layer between the external and internal regions. In the external region, Fe^3+^ ions are characterized by different magnetic properties due to the lack of adjacent ions and a new type of bond with organic molecules. Furthermore, Fe^3+^ ions are surrounded by two different types of ions in the transition layer from the core and the surface. Consequently, their properties are also different from the ions in the core of the nanoparticles. In all patterns, one can see these sextets marked in Table 3 as S4, S5, S6 and S7. The existence of these sextets has also been confirmed and described recently [11,31].

All spectra also contained a single line (L). As can be seen, the contribution from the L component was the highest for nanoparticles synthesized using ClT and those from Fe_3_O_4_ and Fe_3_O_4_–PPh_3_ NPs were the same. This component could arise from the cation vacancies and some superparamagnetic fine particles with no magnetic hyperfine splitting due to a lack of proper magnetic interactions. On the other hand, the analysis of the S1 sextets allowed us to state that the highest content of the oxidized layered had magnetite synthesized without any modifiers and Fe_3_O_4_–en NPs. The lowest contribution from γ-Fe_2_O_3_ was noted for Fe_3_O_4_–ClT and Fe_3_O_4_–PPh_3_ NPs. However, the high contribution from the single line suggests other non-magnetic particles in the sample synthesized in the presence of ClT.

### 3.2. Dielectric Properties of Magnetite Nanoparticles with Different Sizes, Shapes, and Maghemite Contents

**Figure 5:**
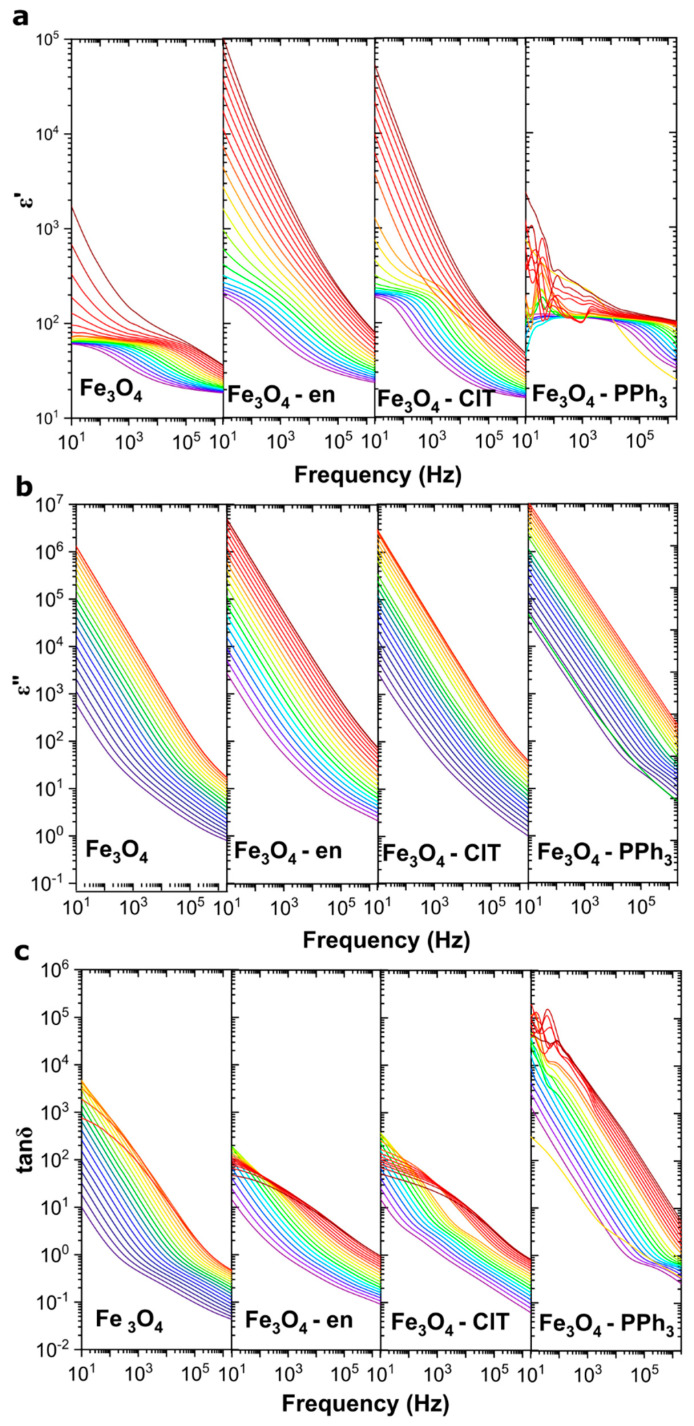
Dielectric properties of magnetite nanoparticles with different shapes and oxidized surfaces: real (**a**) and imaginary (**b**) parts of permittivity, (**c**) loss tangents in different temperatures from 173 K (violet line) to 363 K (red line) with ΔT = 10 K.

For all the synthesized samples, the dielectric properties of Fe_3_O_4_@γ-Fe_2_O_3_ nanoparticles with a core–shell structure were measured as a function of frequency and temperature. The obtained dielectric spectra, i.e., the real and imaginary parts of electric permittivity and the loss tangent, are presented in Figure 5. Complex electric permittivity ε* can be expressed as *ε*′ + *iε*″, and the loss factor tanδ can be described as the ratio of *ε*″/*ε*′. ε* was measured by the dielectric spectroscopy indirectly by measuring complex capacitance C* and calculated by Equation (1) [32,33]:

C* = ε*ε_0_*A*/*h*, (1)

where *ε*_0_ is the vacuum permittivity (8.85 × 10^−12^ As/(V·m)), *A* is the surface area, and *h* is the height of the measured sample.

In Figure 5, the electrical permittivity characteristics for various ferrites are shown. At low frequencies (10 Hz) and low temperatures, it is possible to determine the plateau region (in which *ε*′ was nearly frequency-independent and can be marked as *ε*′*_const_*) for magnetite nanoparticles containing high γ-Fe_2_O_3_ content. The *ε*′*_const_* for Fe_3_O_4_ NPs was equal to 61, that for Fe_3_O_4_–en NPs was 192, and that for Fe_3_O_4_–ClT NPs was 185, all of which increased with increasing temperature. For Fe_3_O_4_–PPh_3_ NPs, it is impossible to study the low-frequency *ε*′, especially in high-temperature regions. It is known, from the structural analysis, that these nanoparticles were cuboidal and had relatively low maghemite content.

The presence of the plateau region and the *ε*′ dispersion in the high-frequency range is associated with the Maxwell–Wagner polarization, which is characteristic of inhomogeneous samples, such as highly conductive particles covered with a resistive surface. The analysis of only the low-frequency region can allow observing that the polarization phenomenon in these nanoparticles is related to the interfacial polarization [34]. As the temperature rises, much more charge carriers accumulate on the surface of the particles, and this polarization is clearly visible. Interestingly, at high temperatures, the role of the magnetite shape, maghemite content, presence of organic molecules, cations vacancies, and surface disordering is very important. For example, at 353 K and 10 Hz, *ε*′ for spherically shaped Fe_3_O_4_ NPs was equal to 329, whereas that for cuboidally shaped Fe_3_O_4_–en NPs was 53,409. Accordingly, the accumulation of electrons increased with the introduction of the maghemite layer onto the magnetite surface and by crystallizing nanoparticles into cuboidal particles with a highly disordered surface.

Interestingly, for Fe_3_O_4_–PPh_3_ NPs with a low γ-Fe_2_O_3_ content, the possibility of the electrons accumulation on the surface of the particles was negligible. In the low-frequency region, only the fluctuation of *ε*′ can be observed, which is related to the high mobility of electrons and high conductivity in this sample, which will be discussed later. The question is: why does the change of shape and surface chemical composition increase the *ε*′ value so drastically? When analyzing this type of nanoparticles, it can be seen that the low-frequency electron transport did not occur from the surface of one nanoparticle to the surface of the second one, but through the disordered γ-Fe_2_O_3_ layer. Accordingly, the accumulation of charge carriers occurred on highly defected, more resistive γ-Fe_2_O_3_ surfaces containing many different defects such as iron vacancies and dislocations [4,14].

The real part of the permittivity is related to the degree of polarization, while the imaginary one provides information about dielectric losses. The *ε*″(*f*) behavior for a wide temperature range from 173 to 363 K is shown in Figure 5b. As can be seen, *ε*″ values are similar for all samples; however, in the low-temperature range, much higher values were observed for the Fe_3_O_4_–PPh_3_ sample. The analysis of dielectric losses can be carried out in the context of the loss tangent, which can represent the energy dissipation in the material under the influence of an applied AC electromagnetic field and for various applications such as supercapacitors should be minimized [35]. In general, increasing the defect density results in higher tanδ values, which have been confirmed in the literature [36]. These defects can be related to the presence of impurities and any imperfections in the crystalline structure.

Interestingly, tanδ did not depend on the maghemite concentration but is closely related to the average crystallite size. Higher dielectric losses were observed for nanoparticles with *D_H-W_* equal to 8.04 nm (Fe_3_O_4_–PPh_3_) and the lowest was identified for Fe_3_O_4_–en NPs with an average crystallite size equal to 11.8 nm. Thus, the storage properties were strongly related to the disordering and oxidation of the magnetite surface and their dissipation properties with their size.

### 3.3. Electrical Conductivity of Magnetite Nanoparticles

The electrical conductivity of magnetite nanoparticles with a modified surface and shape was determined based on measurements of complex electrical conductivity and complex dielectric modulus. The electrical conductivity σ* is related to the electrical permittivity according to Equation (2), and the complex dielectric modulus (M*) is given by Equation (3) [33,37]:

σ* = *σ*′ − *iσ*″ = *i2πfε*_0_ε*, (2)

M* = *M*′ − *iM*″ = 1/ε*. (3)

**Figure 6:**
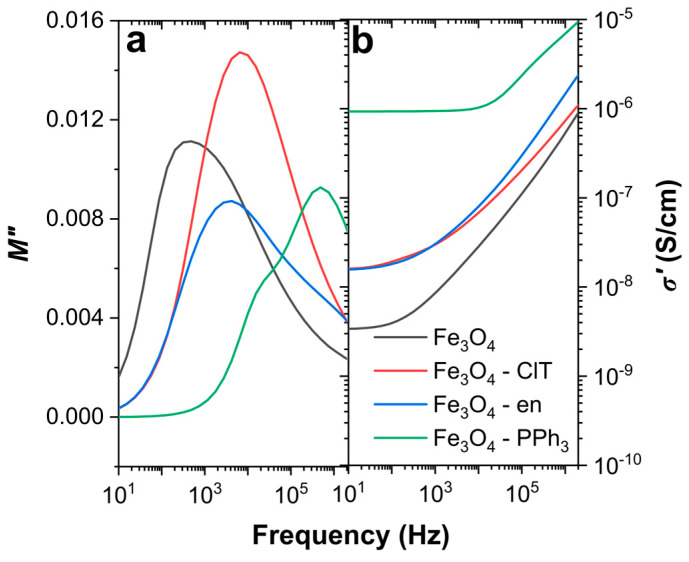
Comparison of electric properties of magnetite nanoparticles at a constant temperature equal to 173 K: (**a**) imaginary part of the dielectric modulus; and (**b**) real part of the electrical conductivity.

The analysis of complex electrical conductivity can provide information about the lag in the movements of charge carriers associated with rapid changes of the electric field and information about their movement, type of electrical conductivity mechanism, and even DC conductivity. The complex dielectric modulus can be used to determine the number of electric processes in materials and the relaxation time of the transition between long- and short-range charge carriers motions (especially in heterogeneous systems) [38]. The imaginary part of the dielectric modulus *M*″, together with the real part of electrical conductivity *σ*′, is presented in Figure 6. As can be seen, both *M*″ and *σ*′ were different for all synthesized nanoparticles. The most visible changes can be observed for the electrical conductivity of Fe_3_O_4_ NPs and Fe_3_O_4_–PPh_3_ NPs. The *σ*′ value at the low-frequency region was a few orders higher for nanoparticles synthesized in the presence of PPh_3_. These nanoparticles were characterized by the low concentration of the maghemite phase, cuboidal shape, and the surface free from organic modifiers. The low-frequency electrical conductivities of Fe_3_O_4_–en and Fe_3_O_4_–ClT NPs were similar and higher than that of pure Fe_3_O_4_ NPs. It is well-known that low-frequency conductivity is closely related to the slow movement of charge carriers through the particle surface and high frequency to the movement of charge carriers in particles. The transition between these processes can be determined based on the analysis of the *M*″ peak [16]. The presence of an oxidized and disordered surface, organic modifiers, and different shapes drastically change the electrical properties, especially in the low-frequency region, where the surface plays a crucial role in the movement of charge carriers. Accordingly, the oxidized surface of Fe_3_O_4_–en NPs and the additional presence of non-magnetic ultrafine particles in Fe_3_O_4_–ClT NPs caused a decrease in the electrical conductivity in the low-frequency region compared to in spherically shaped Fe_3_O_4_–PPh_3_ NPs. The conductivity of the pure Fe_3_O_4_ NPs was the lowest and related to their spherical shape and the highest maghemite content. In addition, the shapes and maximum positions of the *M*″ peak were different for all nanoparticles and were related to the oxidation and functionalization of their surface. The dual nature of the *M*″ peak was visible for Fe_3_O_4_–PPh_3_ NPs, for which the content of the maghemite phase was relatively low. According to previous studies [11], the high-frequency peak is related to the movements of electrons in the magnetite, while the visible shoulder to their movements in the maghemite. For cuboidal Fe_3_O_4_–ClT NPs containing especially non-magnetic particles, the dual nature of the peak cannot be observed, but for other samples, the presence of broad peaks was related to the overlapping of these two electrical processes.

The modified universal power law (Equation (4)) was used to determine the mechanism of electrical conductivity that occurs in the magnetite nanoparticles with an oxidized and disordered surface. This model, unlike the universal power law, can be used to fit the real and imaginary parts of a complex conductivity simultaneously [17]:

σ* = *σ_DC_* + *A*_1_*ω^n^* − *iA*_2_*ω^n^*, (4)

where *σ_DC_* is the DC conductivity, *A*_1_ and *A*_2_ preexponential factors, and *n* is the exponent, which can be used to determine the electrical conductivity mechanism.

The applicability of this model has recently been validated for magnetite nanoparticles and allowed for a better fit of the theoretical model to the experimental data. The simultaneous fitting of the real and imaginary parts of the electrical conductivity can result in different *n*(*T*) behaviors that can be used to describe the high-frequency behavior. Therefore, this approach should also allow for an accurate and comprehensive analysis of the behavior of the electrons in oxidized magnetite nanoparticles with various shapes. In general, when exponent *n* decreases with increasing temperature, the correlated barrier hopping (CBH) can be used to describe their behavior; when exponent *n* increases with increasing temperature, non-overlapping small polaron tunneling (NSPT) occurs [16]. In some cases, the non-linear behavior of this exponent can be observed. For example, when *n* drops to a minimum value and then increases with increasing temperature, overlapping large polaron tunneling (OLPT) occurs [17]. The quantum mechanical tunneling appears when the *n* value equals 0.8, and only slight changes in its value generated by the temperatures can be observed [39]. Additionally, DC electrical conductivity related to the motion of the electrons through the surface of particles in the low-frequency region can be determined from the modified universal power law. In general, this process is highly temperature-dependent, and the activation energy can be estimated based on the Arrhenius law [40].

**Figure 7:**
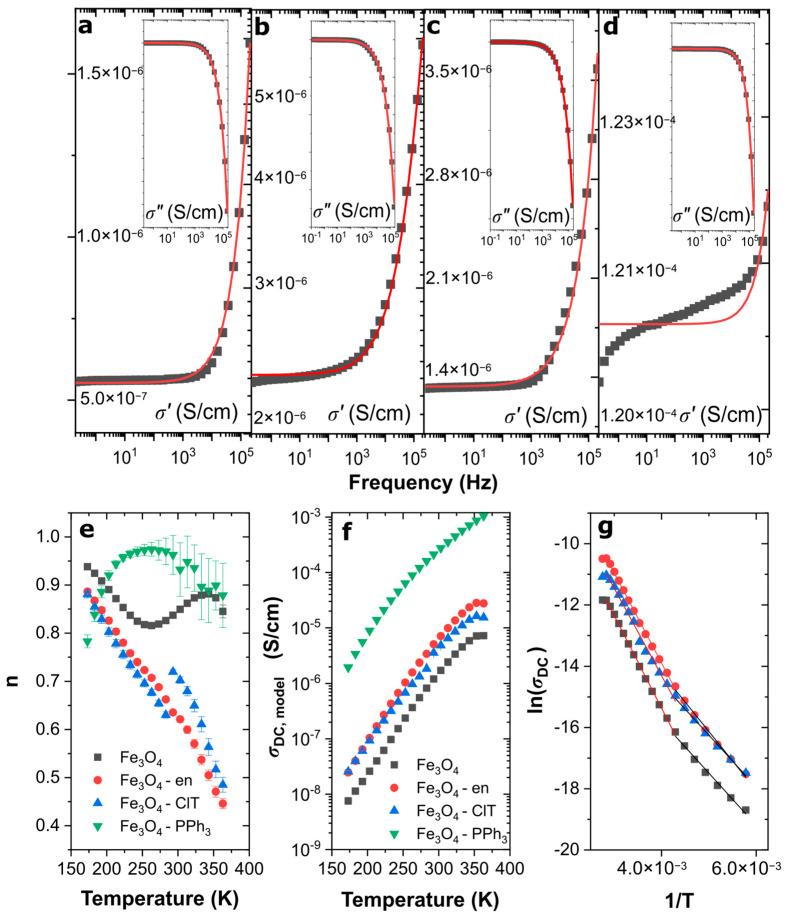
Electrical conductivity for magnetite nanoparticles with different shapes and oxidized surfaces: fitting of the modified universal power law (red line) to the experimental data (black points) for Fe_3_O_4_ NPs (**a**), Fe_3_O_4_–en NPs (**b**), Fe_3_O_4_–ClT NPs (**c**), and Fe_3_O_4_–PPh_3_ NPs (**d**). The insets shows the imaginary part of the electrical conductivity; (**e**) the *n*(*T*) behavior in temperature function with marked measurement errors; (**f**) changes in *σ_DC_* value generated by the temperature changes; (**g**) Arrhenius plots with the fitted curves confirming the existence of two different electrical processes.

Figure 7a–d shows the real and imaginary parts of the electrical conductivity at 273 K, along with the theoretical curves obtained from the fitting by Equation (4). As can be seen, the modified universal power law can be successfully used to describe the complex electrical conductivity over the entire frequency range for samples with relatively low electrical conductivity. Only for Fe_3_O_4_–PPh_3_ NPs, the applicability of this model is limited. However, despite the fact that the modified universal power law cannot describe the real part of electrical conductivity, the imaginary one was very well described by the proposed model.

The model and experimental data differences are strongly related to the ultrafast electron transport between ultrafine magnetite nanoparticles with low maghemite content and cuboidal shape. When for other samples, there was an accumulation and slow motion of electrons on the disordered and oxidized surface (which was discussed in the context of electric permittivity) for Fe_3_O_4_–PPh_3_ NPs, electrons can be easily transferred through the particles surface to the electrodes, which is manifested in a significant drop in *σ*′ at high temperatures and in a low-frequency region. It is also visible in the difference between the values of the low-frequency conductivity for the analyzed samples. For example, *σ*′ equaled 1.33 × 10^−6^ S/cm (at 10 Hz) for Fe_3_O_4_–ClT NPs, whereas *σ*′ for Fe_3_O_4_–PPh_3_ NPs was 100 times higher (1.02 × 10^−4^ S/cm at 10 Hz). Therefore, the calculation of the *n*(*T*) behavior for Fe_3_O_4_–PPh_3_ NPs was burdened with a significant error, especially in the case of high temperatures, which can be observed in Figure 7e. On the other hand, in a low-temperature region, where the mobility of electrons in magnetite was low, the *n*(*T*) behaviors for these particles were characteristic of the NSPT model, which was also theoretically confirmed using DFT + U and hybrid functional calculations [41].

At high temperatures, the ultrafine cuboidal nanoparticles with *n* ≈ 1 should behave similarly to the ideal Debye dielectric dipolar-type crystal. However, the other situation arose in the *n*(*T*) analysis for oxidized samples. While the surface of the oxidized nearly spherical nanoparticles (Fe_3_O_4_ NPs) was free of organic molecules, OLPT occurred. However, correlated barrier hopping occurred when nanoparticles crystallized into cuboidal forms, and organic molecules functionalized their oxidized surfaces (such as ClT and en).

Figure 7f shows the relationship between the DC electrical conductivity obtained from the modified universal power law and temperature. While the *σ_DC_* values for spherically shaped and highly oxidized samples can be satisfactorily analyzed, the *σ_DC_* for Fe_3_O_4_–PPh_3_ NPs was presented only illustratively, because in this type of material, the experimental data did not stay in good agreement with the theoretical model (see Figure 7d). However, with increasing temperature, an increasing tendency of electrical conductivity can be observed for all materials. It is related to the mobility of electrons, which is strongly dependent on temperature. Moreover, it is possible to determine activation energies *E_a_* of electrons moving through the surface of particles. Accordingly, Figure 7g shows the Arrhenius plots for the oxidized samples. The Fe_3_O_4_–PPh_3_ NPs were not analyzed, because the obtained values had a high error. Interestingly, all analyzed samples were characterized by two different activation energies, in low- (173–233 K) and high- temperature (243–363 K) ranges. The calculated values of *E_a_* are listed in Table 4. As one can see, the values of both processes were similar for all nanoparticles (Δ*E_a_* was not higher than ±0.02 eV), because the movement of the charge carriers through the surface occurred in γ-Fe_2_O_3_ and it did not depend on the shape, size, and functionalization. It is well-known that the transport of electrons in magnetite is much easier than in maghemite and is related to the electrons hopping between Fe^3+^ and Fe^2+^ ions [41]. When the magnetite surface is blocked by other non-magnetic nanoparticles, high disordering, or γ-Fe_2_O_3_, in which only Fe^3+^ and iron vacancies exist, the electron movement becomes slower [4,14,42]. In high-temperature regions, movement of electrons increases; however, negatively charged cation vacancies and thermal vibrations of the lattice structure appear. Accordingly, the energy required for this process is much higher than for the low-energy region, in which conductivity is much lower, and electrons can slowly move through the particle surface. 

Based on the comprehensive analysis of the structure and properties of magnetite nanoparticles synthesized in a hydrophobic–hydrophilic environment performed above, it can be concluded that there is a possibility to synthesize Fe_3_O_4_ NPs with determined properties. For example, when good magnetic properties could characterize the nanoparticles, we can adjust the synthesis conditions to maintain these properties and obtain nanoparticles that are excellent electrically conductive (Fe_3_O_4_–PPh_3_ NPs) or have a high ability to store charges on their surface (Fe_3_O_4_ NPs). Both samples have similar and high *M_s_* and low *H_c_* values; however, the electrical conductivity of spherically shaped Fe_3_O_4_ NPs can be even 1000 times lower than Fe_3_O_4_–PPh_3_ NPs at low temperature and in a low-frequency range. However, when the magnetite nanoparticles are used as a capacitor, Fe_3_O_4_–en and Fe_3_O_4_–ClT NPs should be used according to their high ε’ and low tanδ values in wide temperature and frequency ranges [35].

## 4. Conclusions

The surface modification of magnetite plays a key role in synthesizing nanoparticles with specific properties, such as catalytic activity, biocompatibility, and magnetic and dielectric properties. Herein, the possibility of synthesizing ultrafine (9.6 ± 2.0) cuboidal nanoparticles with a relatively low content of an oxidized layer using PPh_3_ as an organic modifier have been confirmed. For these nanoparticles, the highest electrical conductivity (about 1000 times higher than for spherically shaped nanoparticles) and the best superparamagnetic properties (*M_s_* equal to 55.2 emu/g and *H_c_* equal to 8.7 Oe) have been observed. Furthermore, it was confirmed that the transformation of magnetite into maghemite and the copresence of ultrafine non-magnetic nanoparticles caused a decrease in saturation magnetization (44.75 emu/g for the Fe_3_O_4_–ClT NPs). Interestingly, the electric energy storage properties are strictly related to surface disordering and composition. Therefore, *ε*′ was the highest for Fe_3_O_4_–en NPs and Fe_3_O_4_–ClT NPs, for which the oxidized layer, surface disordering and presence of non-magnetic particles or cation vacancies were observed.

On the other hand, dielectric losses are not attributed to an oxidized layer and can be connected with the size of particles. The analysis of the electrical conductivity and the imaginary part of the electric modulus confirmed the high impact of the oxidized surface on the electron behavior. In the *M*″ spectra, a double peak related to the transition between two electric processes has been observed. Moreover, applying the modified universal power law allowed determining the DC conductivity, the activation energy of electron movements through the surface, and the electrical conductivity mechanism. Interestingly, only in the case of Fe_3_O_4_–PPh_3_ NPs, the application of this model was not possible, especially in regions of high temperature. However, the electron behavior and electrical conductivity for Fe_3_O_4_–PPh_3_ NPs can be described using non-overlapping small polaron tunneling in a low-temperature range.

In contrast, at higher temperatures, the behavior of this sample is similar to the ideal Debye dielectric dipolar-type crystal. The electrical conductivity mechanism is also different for nanoparticles with variable γ-Fe_2_O_3_ contents and with a different shape. For Fe_3_O_4_–en NPs and Fe_3_O_4_–ClT NPs, the high-frequency electrical conductivity mechanism is related to the CBH model, whereas for Fe_3_O_4_ NPs it is associated with OLPT model. Accordingly, the presented results can be used to synthesize magnetite nanoparticles with defined magnetic and dielectric properties by modifying the synthesis route. As confirmed, it is simple to prepare Fe_3_O_4_ nanoparticles with similar magnetic properties, whereas their electrical conductivity can differ by a few orders. Moreover, further research should develop restrictions needed at the synthesis step to obtain materials for chosen applications.

## Figures and Tables

**Table 1:** The average crystallite size (*D_H-W_*), intrinsic strain (*ε*), average particle size (*D_av_*), and characteristic for Fe–O bonds vibrations (*υ*_1_, *υ*_2_, and *υ*_2′_).

Sample	*D_H-W_* (nm)	*ε* (10^−3^)	*D_av_* (nm)	*υ*_1_ (cm^−1^)	*υ*_2_ (cm^−1^)	*υ*_2′_ (cm^−1^)
Fe_3_O_4_	9.06	0	11.1 ± 2.2	444.5	583.6	630.0
Fe_3_O_4_–en	11.8	14.6	10.7 ± 3.8	445.5	582.4	629.6
Fe_3_O_4_–ClT	10	13.11	11.4 ± 2.6	444.5	588.0	631.6
Fe_3_O_4_–PPh_3_	8.04	21.7	9.6 ± 2.0	440.6	580.5	630.6

**Table 2:** Magnetic properties of Fe_3_O_4_ NPs (saturation magnetization (*M_s_*), remanence (*M_r_*), coercivity (*H_c_*), and *M_r_*/*M_s_* ratio).

Sample	*M_s_* (emu/g)	*M_r_* (emu/g)	*H_c_* (Oe)	*M_r_*/*M_s_* (a.u.)
Fe_3_O_4_	55.1	1.4	13.6	25.2 × 10^−3^
Fe_3_O_4_–en	52.5	2.1	24.4	39.4 × 10^−3^
Fe_3_O_4_–ClT	44.7	1.1	13.1	23.7 × 10^−3^
Fe_3_O_4_–PPh_3_	55.2	0.9	8.7	15.8 × 10^−3^

**Table 3:** Hyperfine parameters of the synthesized samples (isomer shift (*I_s_*), quadrupole splitting (*Q_s_*), hyperfine magnetic field (*H*), and relative area (*A*)).

Sample	Parameters	Sextets							Single Line
		S1	S2	S3	S4	S5	S6	S7	L
Fe_3_O_4_	I_s_ (mm/s)	0.36	0.34	0.55	0.48	0.18	-	-	0.26
	Q_s_ (mm/)	0.00	0.01	0.00	−0.01	−0.02			-
	H (T)	47.6	43.2	38.2	30.3	19.9			-
	A (%)	41	25	12	7	7			8
Fe_3_O_4_–en	I_s_ (mm/s)	0.32	0.26	0.56	0.36	0.34	0.18	-	0.17
	Q_s_ (mm/)	0.00	−0.01	0.02	0.00	0.01	0.03		-
	H (T)	47.9	43.9	43.0	37.9	29.5	10.4		-
	A (%)	38	19	10	12	10	8		3
Fe_3_O_4_–ClT	I_s_ (mm/s)	0.37	0.25	0.60	0.39	0.36	0.32	-	0.37
	Q_s_ (mm/)	0.00	−0.02	0.02	0.03	0.02	−0.01		-
	H (T)	47.2	42.8	41.8	35.8	27.3	8.6		-
	A (%)	23	11	9	13	15	12		15
Fe_3_O_4_–PPh_3_	I_s_ (mm/s)	0.37	0.26	0.60	0.35	0.31	0.29	0.29	0.28
	Q_s_ (mm/)	0.00	−0.01	0.00	0.01	0.02	−0.02	−0.02	-
	H (T)	46.9	41.8	41.4	35.4	29.9	23.8	9.7	-
	A (%)	27	12	10	10	11	10	12	8

**Table 4:** Activation energies (*E_a_*) of electrical conductivity in low- (173–233 K) and high-temperature (243–363 K) ranges determined from Arrhenius plots.

Sample	*E_a_,_low T_* (eV)	*E_a_,_highT_* (eV)
Fe_3_O_4_	0.15	0.26
Fe_3_O_4_–en	0.17	0.25
Fe_3_O_4_–ClT	0.15	0.24

## Data Availability

The data presented in this study are available on request from the corresponding authors.
